# Antagonism of Therapeutics

**Published:** 1879-04

**Authors:** 


*The Antagonism of Therapeutic Agents, and What it Teaches.* The essay to which was awarded the Fothergillian Gold Medal of the Medical Society of London, for 1878. By J. MILLER FOTHERGILL, M. D., Edin. Philadelphia: Henry C. Lea, 1878.

This book is well worthy of careful perusal, and will correct some common errors concerning incompatibles or antagonistics in the treatment of disease. By experiments upon animals the author shows the antagonism of aconite and atropia, aconite and digitalis, aconite and strychnia, atropia and prussic acid, belladonna and calabar bean, belladonna and jaborandi, belladonna and morphia, bromal hydrate and atropia, caffine and morphine, calabar bean and strychnia, chloral and calabar bean, chloral and picrotoxine, chloral and strychnia, strychnia and hydrocyanic acid, strychnine and nicotine. The experiments upon which conclusions are based were carefully conducted and are as convincing as experiments upon animals can possibly be made. The essay deserves the prize awarded, and merits careful perusal by the medical profession.

